# Telehealthcare in care homes: the use of falls detectors and bed/chair sensors to enhance the safety and experience of care home residents

**Published:** 2012-06-15

**Authors:** Roz Eccles, Alison Anderson

**Affiliations:** NHS Lothian, UK; East Lothian Council, UK

**Keywords:** telecare, falls reduction, care homes

## Abstract

**Introduction:**

It is estimated that 20% of unscheduled admissions to hospital with hip fracture are from care homes. However, evidence suggests that admissions can be reduced by increasing levels of proactive care to residents. Our aim was to utilise appropriate telehealthcare equipment to reduce the number of falls in residents. Telecare products are not new but the range of telecare equipment available has been extended and is smarter. Telecare products monitor people at risk, improving their safety and helping them to stay independent for longer. The equipment used was a portable telecare local alarm and telecare sensors including falls detectors, chair occupancy sensors and bed occupancy sensors. A fractured hip costs between £15,000 and £25,000, a bed monitor costs around £300.

**Aims and objectives:**

Care homes now have increasingly complex residents who require increased levels of nursing care, Telehealthcare was chosen as it was felt this would help decrease the nursing input and assist with releasing time to improve care for residents. This feasibility study evolved as it was felt that traditional methods of falls reduction were not effective i.e. visiting care homes and doing education sessions. It was felt this was not effective due to the transient nature of care home staff and difficulties of engaging them in a short session. It was felt that Telehealthcare would be effective as the care home staff would recognise the equipment as easing their work load. Care Home residents are changing and they have increased care requirements, dementia problems are more prevalent and they have more co morbidities.

**Results:**

Early evidence shows on average a 37% reduction in falls. This represents a significant cost reduction to NHS. Quality of care provided is improved as staff can offer intervene more quickly. Residents and staff also report increased confidence with mobility for those residents with falls detectors as they feel safer and reassured knowing they will be found quickly if they have a fall. This supports releasing time to care as routine checks are no longer required. This has proved particularly valuable overnight as residents are no longer disturbed by two hourly checks and staff are freed up for other tasks. Residents also value the increased privacy as there is less routine monitoring required.

**Conclusions:**

Telecare can be an effective way to assist in the overall management of falls for care home residents. Its use should be considered as part of residents overall care needs and as part of a multi-factorial falls risk assessment when used to its full potential it appears to reduce the overall number of falls significantly and its use is valued by staff, residents and their family/carers. For the purposes of this project the Telecare sensors chosen where specifically aimed at their potential to assist with overall falls management for care home residents. It highlights the potential to develop the use of assistive technology as a whole for this group of people, including other telecare sensors (enuresis), environmental control systems and telehealth.

